# Peripheral prion disease pathogenesis is unaltered in the absence of sialoadhesin (Siglec-1/CD169)

**DOI:** 10.1111/imm.12294

**Published:** 2014-07-29

**Authors:** Barry M Bradford, Paul R Crocker, Neil A Mabbott

**Affiliations:** 1 The Roslin Institute and R(D)SVS, University of EdinburghMidlothian, UK; 2 College of Life Sciences, University of DundeeDundee, UK

**Keywords:** complement component C4, macrophage, prion disease, sialoadhesin, spleen, transmissible spongiform encephalopathies

## Abstract

Prions are a unique group of pathogens, which are considered to comprise solely of an abnormally folded isoform of the cellular prion protein. The accumulation and replication of prions within secondary lymphoid organs is important for their efficient spread from the periphery to the brain where they ultimately cause neurodegeneration and death. Mononuclear phagocytes (MNP) play key roles in prion disease pathogenesis. Some MNP appear to facilitate the propagation of prions to and within lymphoid tissues, whereas others may aid their clearance by phagocytosis and by destroying them. Our recent data show that an intact splenic marginal zone is important for the efficient delivery of prions into the B-cell follicles where they subsequently replicate upon follicular dendritic cells before infecting the nervous system. Sialoadhesin is an MNP-restricted cell adhesion molecule that binds sialylated glycoproteins. Sialoadhesin is constitutively expressed upon splenic marginal zone metallophilic and lymph node sub-capsular sinus macrophage populations, where it may function to bind sialylated glycoproteins, pathogens and exosomes in the blood and lymph via recognition of terminal sialic acid residues. As the prion glycoprotein is highly sialylated, we tested the hypothesis that sialoadhesin may influence prion disease pathogenesis. We show that after peripheral exposure, prion pathogenesis was unaltered in sialoadhesin-deficient mice; revealing that lymphoid sequestration of prions is not mediated via sialoadhesin. Hence, although an intact marginal zone is important for the efficient uptake and delivery of prions into the B-cell follicles of the spleen, this is not influenced by sialoadhesin expression by the MNP within it.

## Introduction

Prion diseases (or transmissible spongiform encephalopathies) comprise a unique group of subacute, fatal, infectious neurodegenerative disorders. During prion disease, aggregations of PrP^Sc^, an abnormally folded isoform of the host cellular prion glycoprotein (PrP^C^) accumulate in affected tissues.^1^ Prion infectivity co-purifies with PrP^Sc^ and is considered to constitute the major component of the infectious agent.^2^,^3^ The accumulation of PrP^Sc^ within the central nervous system (CNS) of prion-infected hosts, coincident with neuronal loss, vacuolation (spongiosis) and reactive glial responses in both astrocytes and microglia, are the major neuropathological hallmarks of prion disease. After peripheral exposure, many prion diseases often accumulate and replicate first in a PrP^C^-dependent manner upon follicular dendritic cells (FDC) within the B-cell follicles of secondary lymphoid organs.^4^,^5^ From these tissues prions subsequently invade the CNS via spreading through the peripheral nervous system^6^,^7^ although haematogenous spread cannot be entirely excluded. For some prion diseases this peripheral phase of infection may be obligatory for efficient transmission to the CNS (termed neuroinvasion).

The precise mode by which infectious prions are initially conveyed from the site of exposure to FDC within the B-cell follicles is uncertain; however, a number of cellular and molecular components of the innate immune system have been shown to be involved. FDC characteristically trap and retain native antigen on their surfaces in the form of immune complexes, consisting of antigen–antibody and/or opsonizing complement components. The FDC appear to initially trap and retain prions on their surfaces in the form of complement-bound complexes.^8^–^10^ In addition to these molecular factors, migratory cells such as integrin αx-expressing (Itgax/CD11c) mononuclear phagocytes (MNP) may also play an important role in the initial uptake and transport of prions because their depletion before peripheral exposure blocked prion accumulation upon FDC and reduced disease susceptibility.^11^,^12^ Complement components may also aid the initial uptake of prions by MNP.^13^

The splenic marginal zone (MZ) surrounding the white pulp comprises a marginal sinus with a network of sinus-lining cells and reticular cells through which the blood percolates on its way to the red pulp. Attached to this network are specific populations of MNP and B cells which enable the continual surveillance and clearance of pathogens, antigens and apoptotic cells from the bloodstream. The positioning of these cells in the MZ enables them to regulate the entry of blood-borne immune complexes and proteoglycans into the FDC-containing B-cell follicles.^14^,^15^ For example, in lymph nodes the sub-capsular sinus macrophages capture immune complexes on their surfaces and pass them to follicular B cells for delivery to FDC in the B-cell follicles.^16^ An intact splenic MZ also plays an important role in the efficient delivery of complement-bound prions to FDC.^17^,^18^

Treatments that block the early accumulation and replication of prions in the spleen can block or substantially reduce disease susceptibility.^8^,^11^,^19^,^20^ Therefore, identification of the cellular and molecular factors that influence the initial accumulation of prions in secondary lymphoid tissues may reveal novel targets for therapeutic intervention. Sialic acids are a large family of nine-carbon sugars that are normally found at the terminal, exposed positions of glycans at the cell surface and on secreted proteins. Sialoadhesin can bind to sialic acid residues expressed on the surface of some important pathogens such as porcine reproductive and respiratory syndrome virus, HIV-1, *Trypanosoma cruzi*, *Neisseria meningitides* and *Campylobacter jejuni*.^21^ Host-encoded sialic acid binding immunoglobulin-like lectin (siglec) proteins are expressed by various MNP subsets under steady-state or are induced during inflammatory conditions and act as cell–cell and cell–pathogen recognition and endocytic internalization receptors.^22^ Sialoadhesin (siglec-1/CD169) binds sialylated glycan end chains^23^ and specifically recognizes *N*-acetylneuraminic acid (Neu5Ac), the predominant sialic acid found in mammalian cells.^24^ The prion protein is variably glycosylated and extensively sialylated^25^ and undergoes altered sialylation during disease-associated accumulation in the CNS.^26^ Since MZ metallophilic macrophages (and sub-capsular sinus macrophages in lymph nodes) are characterized by their constitutive expression of sialoadhesin,^27^ in the current study we tested the hypothesis that sialoadhesin plays an important role in regulating the initial delivery of prions to FDC within the B-cell follicles.

## Materials and methods

### Mice

Siaoladhesin-deficient (Sn^−/−^) mice^28^ were bred and maintained on a C57BL/6 background. Age-matched (5–7 weeks old) C57BL/6 mice were used as wild-type (WT) controls. Mice were housed under specific pathogen-free conditions with a 12 : 12-hr light : dark cycle and food and water were provided *ad libitum*. All experiments were conducted under the provisions of the UK Animals Scientific Procedures Act 1986 and approved by the University of Edinburgh's Ethical Review Committee.

### Prion infection

Groups of Sn^−/−^ mice and WT mice were injected via either the intracerebral or intraperitoneal route with 20 μl of a 1% (weight/volume) brain homogenate prepared from mice terminally infected with ME7 scrapie prions. Mice were culled at the intervals indicated after exposure, or observed for signs of clinical prion disease as described elsewhere^17^ and culled at a standard clinical end-point. Disease incubation periods were calculated as the interval between injection and positive clinical assessment of terminal prion disease.

### Neuropathological assessment

Clinical prion disease diagnosis was confirmed by histopathological assessment of vacuolation (spongiform pathology) in the brain. Brain tissues were fixed in 10% formal saline for a minimum of 48 hr, then processed and embedded in paraffin. Sections (6 μm in thickness) were cut and stained with haematoxylin & eosin and scored for spongiform vacuolar degeneration. For the construction of lesion profiles, vacuolar changes were scored in several grey matter and white matter areas of each brain, as described previously.^29^ Briefly, mouse brain coronal sections were scored for the presence and severity (scale 0–5) of prion-disease-specific vacuolation in nine grey matter and three white matter brain areas: G1, dorsal medulla; G2, cerebellar cortex; G3, superior colliculus; G4, hypothalamus; G5, medial thalamus; G6, hippocampus; G7, septum; G8, cerebral cortex; G9, forebrain cerebral cortex; W1, cerebellar white matter; W2, midbrain white matter; W3, cerebral peduncle.

### Passive immunization

To assess antigen trapping *in vivo*, mice were passively immunized by intravenous injection with 100 μl pre-formed peroxidase–anti peroxidase (PAP) immune complexes (Sigma, St Louis, MO) as described^4^ or 70 000 molecular weight dextran-FITC (Sigma) as described previously.^30^ To prepare fluorescently labelled PrP^Sc^ (Alexa-PrP^Sc^), PrP^Sc^ was enriched from the brains of mice terminally affected with ME7 scrapie prions and labelled as described previously.^31^,^32^

### Immunohistochemical analysis

Tissues were fixed in periodate–lysine–paraformaldehyde fixative and embedded in paraffin wax. Sections (thickness 6 μm) were deparaffinized, pre-treated by autoclaving in water at 121° for 15 min, and immersed in 98% formic acid for 10 min. Sections were incubated overnight with primary antibody. For the detection of astrocytosis, rabbit anti-glial fibrillary acidic protein (Dako, Glostrup, Denmark); for microgliosis, rabbit anti-AIF1/Iba1 (Wako, Richmond, VA); for disease-specific PrP (PrP^d^) in brain, mouse anti-PrP clone 6H4 (Prionics, Schlieren-Zurich, Switzerland); and for spleen, rabbit polyclonal anti-PrP 1B3.^33^ Antibody binding was detected using biotinylated goat anti-species specific antibodies (Jackson Immunoresearch, West Grove, PA) and visualized using the Elite ABC/HRP kit (Vector Laboratories, Peterborough, UK) and diaminobenzidine (DAB) or NovaRed (Vector Laboratories) between stringent washing steps. Sections were lightly counterstained with haematoxylin and imaged on an E800 microscope (Nikon) using image pro plus (Media Cybernetics, Rockville, MD) software. Paraffin-embedded tissue (PET) immunoblot analysis was used to confirm that the PrP^d^ detected by immunohistochemistry (IHC) was prion disease-specific proteinase K (PK)-resistant PrP^Sc^.^34^

For the detection of other markers, additional immunostainings were performed on fixed or frozen sections using the following antibodies: biotin anti-mouse MAdCAM-1 (clone MECA-367; BioLegend, San Diego, CA); CD45RO-B220:allophycocyanin (Clone RA3-6B2; BioLegend); CD169:Alexa488 (Clone MOMA-1; AbD Serotec, Raleigh, NC); rat anti-C4 (Clone FDC-M2; AMS Biotechnology, Abingdon, UK); rat anti-CD21/35 [CR1/CR2] (clone 7G6; BD Pharmingen, Franklin Lakes, NJ). Rat anti-CD35 [CR1] (clone 8C12; BD Pharmingen); Armenian hamster anti-CD209b/SIGNR-1 (clone 22D1; eBioscience, San Diego, CA) or rat anti-SIGNR-1:Alexa647 (clone ER-TR9; AbD Serotec); rat anti-MARCO (clone ED31; AbD Serotec); rabbit polyclonal anti-PrP 1B3; PAP complexes were visualized using AlexaFluor 488-conjugated goat anti-rabbit IgG. The following species-specific secondary antibodies, were used: anti-Armenian hamster, anti-rabbit and anti-mouse all raised in goat or streptavidin; all Alexa-Fluor 488, -594 or -647 conjugates (Invitrogen, Paisley, UK); DyLight™ 649 goat anti-rat IgG (Clone Poly4054; Biolegend). Sections were mounted in fluorescent mounting medium (DakoCytomation, Glostrup, Denmark) and examined using a Zeiss LSM5 confocal microscope (Zeiss). Image analysis was performed using imagej software (http://imagej/nih.gov/ij) on a minimum of six animals per group and six observations per animal, for 72 individual images analysed per comparison.

### Statistical analysis

Statistical analyses were performed using minitab 16 software (Minitab Ltd., Coventry, UK). Incubation period and immunofluorescence analysis quantification data were tested for equal variances and analysed by two-sample *t*-test. Vacuolation profile data were analysed via analysis of variance and grouped via Tukey's post hoc testing.

## Results

### Effect of sialoadhesin-deficiency on FDC status and function

**Figure 1:**
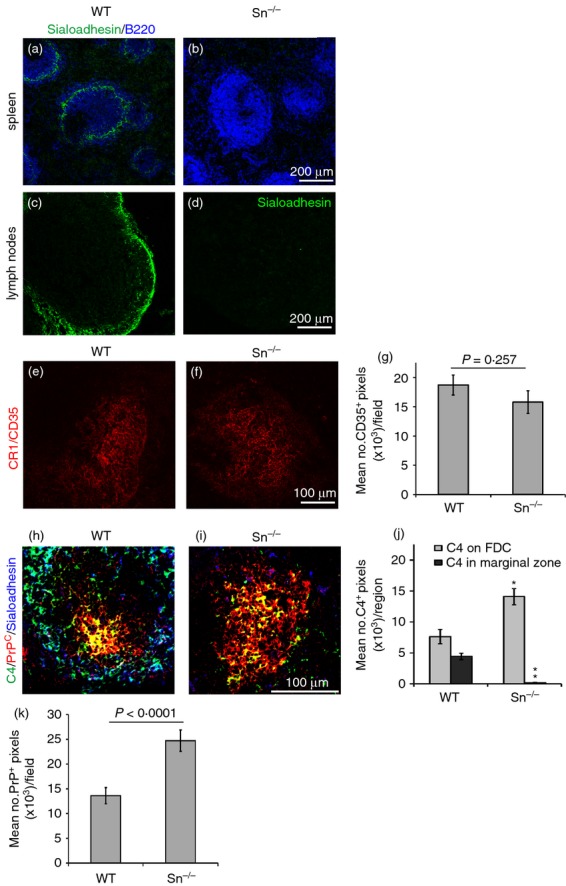
Effect of sialoadhesin-deficiency on follicular dendritic cell (FDC) status. Splenic marginal zone (a) and lymph node sub-capsular sinus macrophages (c) express high levels of sialoadhesin (green) in wild-type (WT) mice, whereas sialoadhesin is undetectable in the spleen (b) and lymph nodes (d) of sialoadhesin-deficient (Sn^−/−^) mice. Sections were also immunostained to detect B cells (B220^+^ cells, blue). Immunostaining with anti-CD35 (e, f, red) revealed that sialoadhesin-deficiency had no effect on FDC network size (g). Comparison of sialoadhesin (blue), complement component C4 (green) and PrP^C^-expression (red) in the spleens of WT (h) and Sn^−/−^ (i) mice. In the spleens of Sn^−/−^ mice, morphometric analysis revealed significant increases in the level of complement component C4 on the surfaces of FDC (j) and their expression of PrP^C^ (k) when compared with WT mice. *P <* *0·001; **P <* *0·0001.

In the spleens of WT mice, high levels of sialoadhesin expression were detected only in association with the inner MZ metallophilic macrophages surrounding the white pulp (Fig. 1a) and their counterparts, the sub-capsular sinus macrophages, in lymph nodes (Fig. 1c). As anticipated, these MNP populations lacked sialoadhesin expression in tissues from sialoadhesin-deficient mice (Fig. 1b,d). As prion replication upon PrP^C^-expressing FDC is important for efficient neuroinvasion, we next determined whether sialoadhesin-deficiency affected FDC status or function. FDC characteristically express high levels of complement receptor 1 (CR1/CD35) on their surfaces. IHC analysis suggested that sialoadhesin-deficiency had no observable effect on the expression of CR1 by FDC in the spleen (Fig. 1e,f). Morphometric analysis also suggested that size of the FDC networks was similar in the spleens of mice from each group (Fig. 1g, *n* = 6 mice per group, *P *=* *0·257). These data are consistent with the expression of negligible levels of the *Siglec1* gene (which encodes sialoadhesin) by FDC in contrast to MNP (see Supporting information, Fig. S1).

The FDC trap and retain complement component C4 on their surfaces and express high levels of cellular PrP^C^ (Fig. 1h). These characteristics are important for the retention and replication of prions upon the surfaces of FDC.^4^,^8^–^10^ IHC revealed that significantly more complement component C4 was detected on the surfaces of FDC in the spleens of Sn^−/−^ mice when compared with WT controls (Fig. 1i,j; *n *=* *6 per group, *P *<* *0·001). This coincided with a significant reduction in the ability of cells in the MZ of sialoadhesin-deficient mice to trap complement component C4 (Fig. 1i,j, *n* = 6 mice per group, *P *<* *0·0001). Data suggest that the trapping of immune complexes by FDC up-regulates their expression of PrP^C^.^35^ Here, the increased association of C4 on the surfaces of FDC in the spleens of Sn^−/−^ mice coincided with a significant increase in the expression of PrP^C^ upon their surfaces (Fig. 1k; *n *=* *6 mice per group, *P *<* *0·0001) implying a similar relationship.

**Figure 2:**
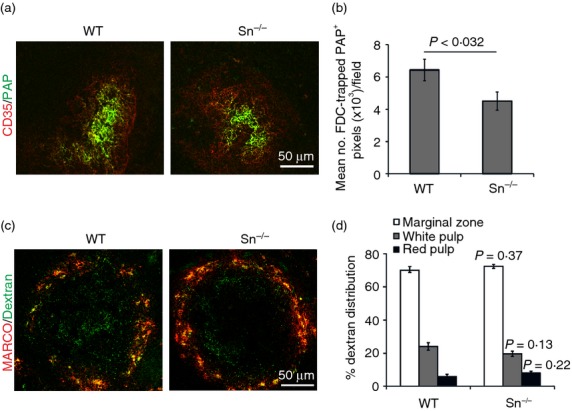
Effect of sialoadhesin-deficiency on passive immune complex trapping upon follicular dendritic cells (FDC) and lectin-mediated endocytic uptake in the marginal zone (MZ). (a, b) Effect of sialoadhesin-deficiency on the ability of FDC to trap pre-formed peroxidase–anti-peroxidase (PAP) immune complexes. Sections were immunostained to detect FDC (CD35^+^ cells, red) and PAP (rabbit IgG, green). (c, d) The trapping of dextran-FITC within the splenic MZ is unaltered in sialoadhesin-deficient (Sn^−/−^) mice.

To determine whether sialoadhesin-deficiency influenced the trapping and retention of immunoglobulin/antigen-containing immune complexes by FDC, mice from each group were passively immunized with PAP immune complexes, and 24 hr later, the presence of FDC-associated immune complexes was identified by IHC (Fig. 2a). Although FDC from each group retained high levels of PAP immune complexes, a small but significant reduction was observed in the spleens of Sn^−/−^ mice (Fig. 2b; *n *=* *6 mice per group, *P *=* *0·032).

### Effect of sialoadhesin-deficiency on the lectin-mediated endocytic uptake of polysaccharides by MZ macrophages

Mononuclear phagocytes in the MZ play important roles in the uptake and clearance of particulate antigens from the bloodstream. Situated within the outer layer of the MZ are a ring of macrophage receptor with collagenous structure (MARCO) -expressing MZ macrophages, whereas the sialoadhesin-expressing MZ metallophilic macrophages form a continuous inner ring close to the white pulp. MARCO-expressing MZ macrophages also express an array of scavenger receptors including scavenger receptor-A (SR-A) and the C-type lectin SIGNR1,^14^ which recognize specific carbohydrate structures such as bacterial capsular polysaccharides. We next determined whether the phagocytosis of polysaccharide antigens by MARCO-expressing MZ macrophages was influenced by sialoadhesin-deficiency. Mice from each group were passively immunized with fluorescently labelled dextran particles (dextran-FITC) and spleens were analysed 1 hr later by IHC. No differences in MARCO expression by MZ macrophages or in their ability to endocytose dextran within the MZ were observed between spleens of WT and Sn^−/−^ mice (Fig. 2c). In spleens from each mouse group the majority of the dextran-FITC was typically co-localized with MARCO-expressing macrophages within the outer layer of the MZ (Fig. 2d) with lesser amounts being observed within the red pulp and occasionally within follicular B-cell areas as was described previously.^30^ These data demonstrate that sialoadhesin-deficiency does not affect the ability of MARCO-expressing MZ macrophages to clear blood-borne dextran particles (Fig. 2d).

### Effect of sialoadhesin-deficiency on the early accumulation of PrP^Sc^ in the spleen

Within weeks after peripheral exposure, ME7 scrapie prions accumulate first upon FDC in the secondary lymphoid organs and are maintained at high levels for the duration of the disease. We next determined the effect of sialoadhesin-deficiency on the initial accumulation of prions upon FDC in the spleen. In this study, the normal cellular form of the prion protein is referred to as PrP^C^ and two distinct terms (PrP^Sc^ and PrP^d^) are used to describe the disease-specific, abnormal accumulations of PrP that are characteristically found only in prion-affected tissues. Prion disease-specific PrP accumulations are relatively resistant to PK digestion, whereas cellular PrP^C^ is destroyed. As a consequence, PK-resistant PrP (referred to as PrP^Sc^) can be used as a biochemical marker for the presence of prions.^36^ Since PK-treatment is not suitable for use on histological sections, we refer to these abnormal, disease-specific PrP accumulations as PrP^d^. On histological sections, to confirm the presence of prion-specific PK-resistant PrP^Sc^, adjacent tissue sections were applied to nitrocellulose membrane, treated with PK, and analysed by PET immunoblot.^34^

**Figure 3:**
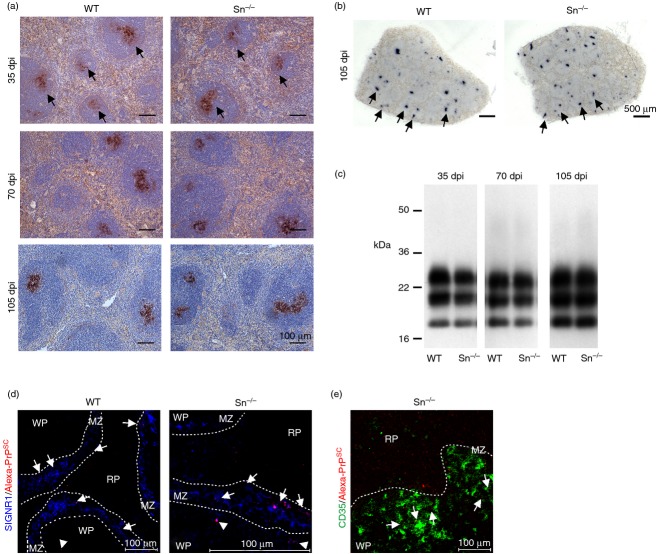
Effect of sialoadhesin-deficiency on prion accumulation in the spleen. (a) Immunohistochemical detection of prion-specific abnormal PrP (PrP^d^, brown, arrows) upon follicular dendritic cell (FDC) in the spleens of wild-type (WT) and sialoadhesin-deficient (Sn^−/−^) mice. Sections counterstained with haematoxylin (blue). dpi, days post-injection with prion. (b) Paraffin-embedded tissue immunoblot analysis confirmed the presence of prion-specific, proteinase K (PK) -resistant PrP^Sc^ (black) upon FDC (arrows). (c) Immunoblot analysis of PK-treated spleen tissue homogenates confirmed the accumulation of high levels of prion-specific, PK-resistant PrP^Sc^. After PK treatment, a typical three-band pattern was observed between molecular mass values of 20–30 000 MW, representing unglycosylated, monoglycosylated and diglycosylated isomers of PrP (in order of increasing molecular mass). Approximate molecular weight markers are indicated. (d, e) Analysis of spleen sections from Alexa-PrP^Sc^-injected mice suggested that the PrP^Sc^ (red) was mostly associated with SIGNR1^+^ marginal zone (MZ) macrophages (d, blue, arrows), and occasionally in the red pulp (RP) and white pulp (WP; d, arrowheads) in regions in close association with CR1/CD35-expressing (green) FDC (e, arrows). No apparent differences were observed between tissues from WT or Sn^−/−^ mice. Dashed lines indicate the border of the MZ.

Mice were injected intraperitoneally with ME7 scrapie prions and tissues were collected at intervals after exposure. Heavy PrP^d^ accumulations, consistent with localization upon FDC, were detected by IHC in the spleens of all WT mice at 5 weeks after prion exposure (Fig. 3a). By 10 and 15 weeks after intraperitoneal injection with prions, the levels of PrP^d^ upon the surfaces of FDC appeared to have increased, and were maintained for the duration of the disease. PET immunoblot analysis of adjacent tissue sections (Fig. 3b) and immunoblot analysis of tissue homogenates (Fig. 3c) confirmed that the PrP^d^ detected in these tissues was prion disease-specific PrP^Sc^. Similarly, high levels of PrP^Sc^ were also detected in association with FDC in the spleens of Sn^−/−^ mice analysed at the same times after exposure to prions (Fig. 3). These data show that sialoadhesin-deficiency did not impair the early accumulation of PrP^Sc^ upon FDC.

A parallel experiment was also performed to determine whether sialoadhesin-deficiency influenced the accumulation of prion-specific PrP^Sc^ within the spleen. Highly PrP^Sc^-enriched, scrapie-associated fibrils were isolated from the brains of clinical prion disease-affected mice and fluorescently labelled as described previously.^31^,^32^ This labelling enabled the visualization of Alexa-PrP^Sc^ in tissues by immunofluorescence confocal microscopy.^31^,^32^ Mice from each group were injected intravenously with Alexa-PrP^Sc^; spleens were collected 1 hr later and immunostained to detect the SIGNR-1-expressing MZ macrophages. No differences in SIGNR-1 expression by MZ macrophages and in the anatomical localization of Alexa-PrP^Sc^ between spleens of WT and Sn^−/−^ mice were observed. The majority of the Alexa-PrP^Sc^ was observed within the MZ (Fig. 3d, arrows), but small amounts were observed in the white pulp (Fig. 3d, arrowheads) occasionally in close association with FDC (Fig. 3e, arrows).

### Effect of sialoadhesin-deficiency on prion disease susceptibility

**Figure 4:**
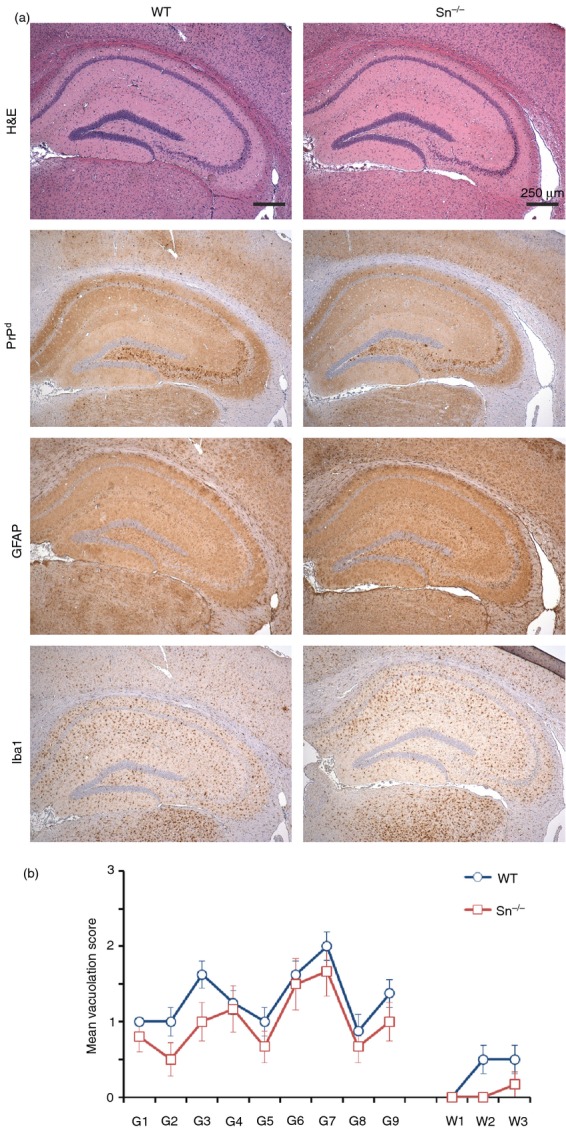
Effect of sialoadhesin-deficiency on the neuropathology in the brains of prion-infected mice. (a) Sialoadhesin-deficiency had no observable effect on disease-specific vacuolation, PrP^d^ deposition, or reactive glial responses in the brains of the clinically affected, peripheral prion-infected mice. High levels of spongiform pathology (haematoxylin & eosin, top row), heavy accumulations of disease-specific prion protein (PrP) (brown, second row), reactive astrocytes expressing glial fibrillary acidic protein (GFAP) (brown, third row), and active microglia expressing Iba1 (brown, bottom row) were detected in the brains of all clinically prion disease-affected wild-type (WT) mice (left-hand column) and sialoadhesin-deficient (Sn^−/−^) mice (right-hand column). (b) Pathological assessment of the spongiform change (vacuolation) in brains from terminally prion-affected WT (blue line) and Sn^−/−^ (red line) mice. Vacuolation was scored on a scale of 0–5 in the following grey (G1–G9) and white (W1–W3) matter areas: G1, dorsal medulla; G2, cerebellar cortex; G3, superior colliculus; G4, hypothalamus; G5, thalamus; G6, hippocampus; G7, septum; G8, retrosplenial and adjacent motor cortex; G9, cingulate and adjacent motor cortex; W1, inferior and middle cerebellar peduncles; W2, decussation of superior cerebellar peduncles; and W3, cerebellar peduncles.

We next determined whether sialoadhesin-deficiency influenced prion neuroinvasion or disease susceptibility. Our data show that sialoadhesin-deficiency had no significant effect on the spread of prions to the brain after intraperitoneal injection as all WT and Sn^−/−^ mice succumbed to clinical prion disease with similar incubation periods (Table 1, *P* = 0·130). Similarly, when WT and Sn^−/−^ mice were injected with prions directly into the CNS by intracerebral injection, they developed clinical prion disease with similar incubation periods (Table 1, *P* = 0·717). Histopathological analysis confirmed that brains from all clinically affected WT and Sn^−/−^ mice displayed the characteristic spongiform pathology, astrogliosis, microgliosis and PrP^d^ accumulation typically associated with terminal infection with ME7 scrapie prions (Fig. 4). Together, these data reveal no significant effect of sialoadhesin-deficiency on prion neuroinvasion or disease susceptibility.

**Table 1:** Effect of sialoadhesin-deficiency on prion disease susceptibility

	Route of prion exposure			
Intracerebral injection		Intraperitoneal injection	
Mouse strain	Incidence of clinical disease^1^	Mean incubation period (days) ± SEM	Incidence of clinical disease	Mean incubation period (days) ± SEM
WT	5/5	153 ± 4	8/8	241 ± 7
Sn^−/−^	6/6	151 ± 4	6/6	256 ± 6

Abbreviations: Sn^−/−^, sialoadhesin-deficient; WT, wild-type.

1 Number of mice affected/injected with prions.

## Discussion

Prion replication upon FDC in secondary lymphoid organs is critical for their efficient neuroinvasion.^4^,^19^,^20^ However, as FDC are non-motile, stromal-derived cells which form networks in the B-cell follicles^37^,^38^ it is uncertain how prions are first conveyed to secondary lymphoid tissues and transferred to FDC. Both cell-associated and cell-free mechanisms have been suggested.^8^–^10^,^39^ The splenic MZ is a specialized microenvironment that plays an important role in the capture and clearance of blood-borne pathogens/antigens. Specific populations of MNP within the MZ (and sub-capsular sinus of lymph nodes) have also been shown to capture immune complexes and present them to follicular B cells for delivery to FDC.^15^,^16^,^40^ The opsonization of prions with complement components is important for their retention by FDC.^8^–^10^ In studies elsewhere we have also shown that an intact MZ is also important for the initial localization of prions upon FDC.^17^,^18^ These data imply that a similar cellular relay may shuttle complement-opsonized prions to FDC. The prion protein is highly sialylated,^25^ and MZ metallophilic macrophages in the spleen, and the sub-capsular sinus macrophages in the lymph nodes, constitutively express high levels of sialoadhesin, which binds to sialylated glycoproteins.^27^ Prions may also be released from infected cells in association with exosomes and play an important role in their cell-to-cell transmission.^41^,^42^ A role for MNP-expressed sialoadhesin in the capture of B-cell-derived exosomes in the spleen and lymph nodes via surface expressed sialic acids has also been described.^43^ The identification of molecular factors that influence the initial accumulation of prions in secondary lymphoid tissues may reveal novel targets for therapeutic intervention. Therefore, in the current study we used Sn^−/−^ mice to test the hypothesis that sialoadhesin-expressing MNP in the MZ play an important role in the initial uptake of prions in the spleen. However, our data show that after peripheral prion disease exposure the early accumulation of PrP^Sc^ on FDC, subsequent neuroinvasion and disease susceptibility were not impaired in Sn^−/−^ mice. Hence, these data show that the efficient delivery of prions to FDC in the spleen is not influenced by sialoadhesin-expression by MZ metallophilic MNP. These data do not preclude the possibility that MZ metallophilic macrophages may be involved in prion uptake and transfer into the B-cell follicles, only that sialoadhesin itself is non-essential for this process.

In WT mice high levels of activated complement component C4 were detected in association with FDC, which is indicative of the retention of complement-opsonized immune complexes on their surfaces.^44^ However, low levels were also detected in association with the MZ. Studies elsewhere show that in the absence of effective immune-complex shuttling from the MZ to FDC, MZ B cells appear unable to ‘off-load’ their complement-opsonized antigens, resulting in their increased accumulation of complement component C4.^15^,^45^,^46^ In the spleens of Sn^−/−^ mice the MZ localization of complement component C4 was significantly reduced. Furthermore, this coincided with a significant increase in complement component C4 upon FDC in the spleens of Sn^−/−^ mice (Fig. 1). Glycan profiling of complement component C4 has revealed that it carries a mixture of both oligomannose and complex bi-antennary glycans with varying amounts of sialylation.^47^ These data suggest that sialoadhesin may play an important role in the initial uptake and sequestration of blood-borne complement-opsonized immune complexes by MZ metallophilic macrophages via terminal sialic acid residues on the glycans on complement component C4. Our data also suggest that in the absence of this activity in Sn^−/−^ mice, less complement component C4 is sequestered by MNP in the MZ, enabling more to be shuttled to FDC. The retention of immune complexes on FDC promotes the ability of naive IgM^+^ B cells to mature and class switch to high-affinity IgG.^48^ Whether the significantly reduced IgM titre in the serum of Sn^−/−^ mice^28^ is a consequence of the effects of sialoadhesin-deficiency on complement component C4 retention is uncertain.

The expression of PrP^C^ by FDC has been shown to be stimulated by their retention of complement-opsonized immune complexes, and this up-regulation in PrP^C^-expression is blocked in mice that are deficient in complement component C1q.^35^ Conversely, the dramatically impaired retention of immune complexes by FDC in aged mice coincides with substantially reduced levels of PrP^C^ expression.^17^,^18^,^49^ In the current study, coincident with the increased accumulation of complement component C4, the level of PrP^C^ expressed by FDC was also significantly increased, implying complement-mediated up-regulation of PrP^C^-expression by FDC.

Prions are considered to be initially acquired by FDC as complement-opsonized immune complexes.^4^,^8^–^10^ In the absence of sialoadhesin expression by metallophilic macrophages the retention of complement component C4 upon FDC was increased. Hence, it is plausible that in the spleens of Sn^−/−^ mice fewer complement-opsonized prions were sequestered by MNP in the MZ, enabling them to be shuttled more efficiently to FDC.

In summary, despite the important role of sialoadhesin in the binding of sialylated antigens^21^–^24^ and exosomes,^43^ our data show that after peripheral prion exposure sialoadhesin expression by metallophilic macrophages in the inner ring of the splenic MZ is not required for the efficient transport of prions to FDC and the subsequent spread of disease to the CNS. Treatments that block the early accumulation of prions upon FDC can block or substantially reduce disease susceptibility.^8^,^11^,^19^,^20^ Therefore, identification of the cellular and molecular factors that influence the initial delivery of prions to FDC in the B-cell follicles of secondary lymphoid tissues may reveal novel targets for therapeutic intervention in peripherally acquired infections, or factors that influence disease susceptibility.

## Abbreviations

CNS: central nervous system
FDC: follicular dendritic cell
IHC: immunohistochemistry
MARCO: macrophage receptor with collagenous structure
MNP: mononuclear phagocyte
MZ: splenic marginal zone
PAP: pre-formed peroxidase–anti peroxidase
PET: paraffin-embedded tissue
PK: proteinase K
PrP: prion protein
Sn^–/–^: sialoadhesin-deficient
WT: wild-type

## Supporting Information

Figure S1 Comparison of *Siglec1* mRNA expression in murine tissues and cells.
