# Supplementary material for: Peripheral prion disease pathogenesis is unaltered in the absence of sialoadhesin (Siglec-1/CD169)
Source: Immunology. 2014 Jul 29;143(1):120–9. doi: 10.1111/imm.12294 (PMC4137961; doi:10.1111/imm.12294)
Supplement: Figure S1 — Comparison of Siglec1 mRNA expression in murine tissues and cells. [file imm0143-0120-sd1.pdf]

Barry M. Bradford, Paul R. Crocker & Neil A. Mabbott

Peripheral prion disease pathogenesis is unaltered in the absence of sialoadhesin (Siglec-1/CD169)

Supplemental Figure 1

Comparison of *Siglec1* mRNA expression in murine tissues and cells.

(A) Expression profile of *Siglec1* (which encodes Sn/CD169; Affymetrix probe set ID 1422161_at) across a wide range of microarray data sets representing 95 distinct mouse tissues and cell-lineages. All sample analysis was performed on the Affymetrix MOE430 2.0 expression array. Data were analysed using the GNF1M Mouse tissue atlas (http://biogps.gnf.org). Detailed conditions of the cell and tissue preparation and treatment are available in the above study. The normalised, mean gene expression level data for each tissue and cell type are shown. These data show that in the steady-state Sn is not expressed by microglia (blue box), Neuro2a cells (green box) or in the brain (red box).

(B) Subcapsular sinus macrophages, in contrast to follicular dendritic cells, express high levels of *Siglec1* mRNA in the steady state. Comparison of *Siglec1* expression levels (Affymetrix probe set 1422161_at) by subcapsular sinus (SCS) macrophages, microglia, FL-Y cells (a follicular dendritic cell (FDC)-like cell-line), Peyer's patches (PP) FDC, astrocytes and neuro2a neuroblastoma cells. Publicly available gene expression data sets performed on Affymetrix MOE430 2.0 expression arrays were download and normalised using RMA (Affymetrix, Santa Clara, CA). The chip identification accession numbers for each data set are indicated: SCS macrophages, GSM395738, GSM395739, GSM395740; microglia, GSM258721, GSM258722; FL-Y-cells, RMLNDC002001, RMLNDC003001; PP-FDC, GSM481976, GSM481977; astrocytes, GSM241898; neuro2a cells, GSM258727, GSM258728.

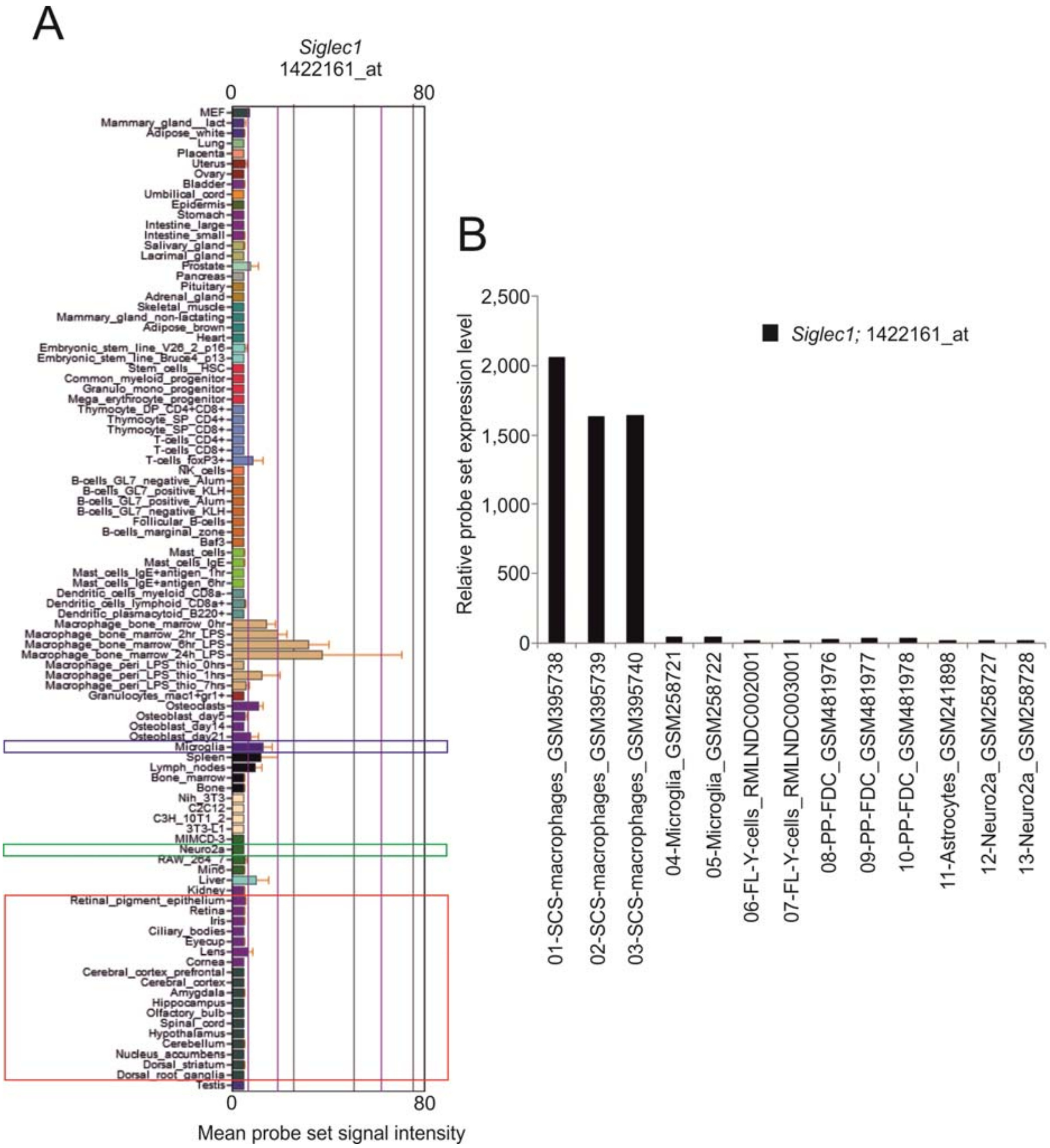
A
Siglec1
1422161_at
0
80
MEF
Mammary_gland__lact
Adipose_white
Lung
Placenta
Uterus
Ovary
Bladder
Umbilical_cord
Epidermis
Stomach
Intestine_large
Intestine_small
Salivary_gland
Lacrimal_gland
Prostate
Pancreas
Pituitary
Adrenal_gland
Skeletal_muscle
Mammary_gland_non-lactating
Adipose_brown
Heart
Embryonic_stem_line_V26_2_p16
Embryonic_stem_line_Bruce4_p13
Stem_cells__HSC
Common_myeloid_progenitor
Granulo_mono_progenitor
Mega_erythrocyte_progenitor
Thymocyte_DP_CD4+CD8+
Thymocyte_SP_CD4+
Thymocyte_SP_CD8+
T-cells_CD4+
T-cells_CD8+
T-cells_foxP3+
NK_cells
B-cells_GL7_negative_Alum
B-cells_GL7_positive_KLH
B-cells_GL7_positive_Alum
B-cells_GL7_negative_KLH
Follicular_B-cells
B-cells_marginal_zone
Baf3
Mast_cells
Mast_cells_IgE
Mast_cells_IgE+antigen_1hr
Mast_cells_IgE+antigen_6hr
Dendritic_cells_myeloid_CD8a-
Dendritic_cells_lymphoid_CD8a+
Dendritic_plasmacytoid_B220+
Macrophage_bone_marrow_0hr
Macrophage_bone_marrow_2hr_LPS
Macrophage_bone_marrow_6hr_LPS
Macrophage_bone_marrow_24h_LPS
Macrophage_peri_LPS_thio_0hrs
Macrophage_peri_LPS_thio_1hrs
Macrophage_peri_LPS_thio_7hrs
Granulocytes_mac1+gr1+
Osteoclasts
Osteoblast_day5
Osteoblast_day14
Osteoblast_day21
Microglia
Spleen
Lymph_nodes
Bone_marrow
Bone
Nih_3T3
C2C12
C3H_10T1_2
3T3-L1
MIMCD-3
Neuro2a
RAW_264_7
Min6
Liver
Kidney
Retinal_pigment_epithelium
Retina
Iris
Ciliary_bodies
Eyecup
Lens
Cornea
Cerebral_cortex_prefrontal
Cerebral_cortex
Amygdala
Hippocampus
Olfactory_bulb
Spinal_cord
Hypothalamus
Cerebellum
Nucleus_accumbens
Dorsal_striatum
Dorsal_root_ganglia
Testis
0
80
Mean probe set signal intensity
B
Relative probe set expression level
2,500
2,000
1,500
1,000
500
0
Siglec1; 1422161_at
01-SCS-macrophages_GSM395738
02-SCS-macrophages_GSM395739
03-SCS-macrophages_GSM395740
04-Microglia_GSM258721
05-Microglia_GSM258722
06-FL-Y-cells_RMLNDC002001
07-FL-Y-cells_RMLNDC003001
08-PP-FDC_GSM481976
09-PP-FDC_GSM481977
10-PP-FDC_GSM481978
11-Astrocytes_GSM241898
12-Neuro2a_GSM258727
13-Neuro2a_GSM258728
